# Cognitive interviews guide design of a new CAM patient expectations questionnaire

**DOI:** 10.1186/1472-6882-14-39

**Published:** 2014-01-25

**Authors:** Karen J Sherman, Emery R Eaves, Cheryl Ritenbaugh, Clarissa Hsu, Daniel C Cherkin, Judith A Turner

**Affiliations:** 1 Group Health Research Institute, 1730 Minor Avenue, Suite 1600, Seattle WA 98101, USA; 2 Department of Family and Community Medicine, University of Arizona, 1450 N Cherry Avenue, Tucson AZ 85719, USA; 3 Center for Community Health and Evaluation, Group Health Research Institute, 1730 Minor Avenue, Suite 1600, Seattle WA 98101, USA; 4 Department of Psychiatry and Behavioral Sciences, University of Washington, Box 356560, Seattle WA 98195-6560, USA

**Keywords:** Cognitive interviews, Questionnaires, Expectations, Low back pain, Acupuncture, Chiropractic, Massage therapy, Yoga

## Abstract

**Background:**

No consistent relationship exists between pre-treatment expectations and therapeutic benefit from various complementary and alternative medicine (CAM) therapies in clinical trials. However, many different expectancy measures have been used in those studies, with no validated questionnaires clearly focused on CAM and pain. We undertook cognitive interviews as part of a process to develop and validate such a questionnaire.

**Methods:**

We reviewed questions about expectations of benefits of acupuncture, chiropractic, massage, or yoga for pain. Components of the questions – verbs, nouns, response options, terms and phrases describing back pain – were identified. Using seven different cognitive interview scripts, we conducted 39 interviews to evaluate how individuals with chronic low back pain understood these individual components in the context of expectancy questions for a therapy they had not yet received. Chosen items were those with the greatest agreement and least confusion among participants, and were closest to the meanings intended by the investigators.

**Results:**

The questionnaire drafted for psychometric evaluation had 18 items covering various domains of expectancy. “Back pain” was the most consistently interpreted descriptor for this condition. The most understandable response options were 0-10 scales, a structure used throughout the questionnaire, with 0 always indicating no change, and 10 anchored with an absolute descriptor such as “complete relief”. The use of words to describe midpoints was found to be confusing. The word “expect” held different and shifting meanings for participants. Thus paired items comparing “hope” and “realistically expect” were chosen to evaluate 5 different aspects of treatment expectations (back pain; back dysfunction and global effects; impact of back pain on specific areas of life; sleep, mood, and energy; coping). “Impact of back pain” on various areas of life was found to be a consistently meaningful concept, and more global than “interference”.

**Conclusions:**

Cognitive interviews identified wordings with considerable agreement among both participants and investigators. Some items widely used in clinical studies had different meanings to participants than investigators, or were confusing to participants. The final 18-item questionnaire is undergoing psychometric evaluation with goals of streamlining as well as identifying best items for use when questionnaire length is constrained.

## Background

Patient expectations of therapeutic benefit are widely thought to be important determinants of treatment outcomes [1-3]. Some studies have suggested that patient expectations of treatment outcomes are one of the most important prognostic factors for patients with back pain [4-6]. However, results from trials of various complementary and alternative medical (CAM) therapies have not consistently supported this belief for musculoskeletal pain [7-10]. The lack of a comprehensive theoretical framework for understanding patient expectations [11] and the paucity of measuring instruments that have been evaluated in relation to participant comprehension and interpretation, reproducibility, and construct validity [3] have impeded progress in understanding how expectations may impact treatment outcomes.

There is disagreement in how best to identify, monitor, and classify patients’ expectations [11,12]. Literature reviews of expectations have reported that when and how expectations are elicited matters [12]. Other studies have reported the importance of distinguishing between “probability expectations” (rational projections) and idealized expectations (hopes) [11]. Cognitive interviews have not been used when developing previously validated questionnaires of treatment expectations [13-15], even though cognitive interviews have been used when developing other questionnaires designed to assess patient reported outcomes [16,17]. A recent document from the United States Food and Drug Administration (FDA) recommends cognitive interviews be conducted when developing patient reported outcomes [18].

Here we report our efforts to develop and validate a comprehensive questionnaire for measuring patient expectations of four CAM treatments commonly used for back pain. We chose to focus on back pain because it is the most common musculoskeletal pain condition and the most common pain condition for which people use CAM care [19]. However, we believe that our questionnaire could be modified slightly to capture expectations for other pain conditions.

After reviewing relevant literature and surveying CAM researchers to identify questions that have been used to assess patient expectations of treatments for back pain, we conducted cognitive interviews with low back pain patients to develop and refine questions and identify issues in measurement of patient expectations. Here we describe the content, structure, and meaning of questions and issues identified in cognitive interviews.

## Methods

### Selection of categories and domains for testing

We used two strategies to identify key components of questions for cognitive testing. First, we conducted qualitative interviews with CAM practitioners regarding their experience with and management of patient expectations [20] and with patients seeking CAM about their treatment expectations. Second, KJS and JT collected extant questions related to participant expectations through two mechanisms: literature searches for published questionnaires on patient expectations; and email queries to acupuncture, chiropractic, massage, and yoga researchers in January of 2010 asking for copies of the questions they used to measure expectations in their trials. (See Additional file 1: Appendix A for researchers contacted, and a representative list of their publications).

Our patient interviews included questions about back pain history, changes they hoped to see as a result of treatment, expectations they had for the treatments and whether these differed from their hopes. During data analyses, we extracted themes related to expectations and outcomes from the treatment, including the desire for diminished pain and better ability to engage in meaningful activities. We then compared themes from interviews with those from existing questionnaires and from a literature review and a drafted a conceptual model of patient expectations [20]. We thereby identified areas of importance not addressed by existing questions. Once relevant themes were elucidated, KJS reviewed the original battery of questionnaires from CAM researchers and published questionnaires and created a document with potentially relevant questions. CR and KJS then reviewed these questions and compared them with themes from the interviews. They found all of the extant questions contained potential ambiguities that required evaluation by cognitive interviews to elucidate participant understanding. We therefore deconstructed the questions and responses into components so we could test terms and phrases related to expectations, outcome domains, timeframes for improvement, and response options. Additional file 1: Appendix A contains a list of researchers who provided us with copies of expectancy-related questions they had used in prior studies. Additional file 1: Appendix B contains a list of representative citations for researchers listed in Additional file 1: Appendix A.

### Cognitive interview methodology

Cognitive interviewing, a prominent method in questionnaire development, is used to detect potential problems with survey questions prior to their widespread use [21-24]. Cognitive interview participants are asked to verbally articulate their thought processes related to selection of responses during or immediately after answering specific questionnaire items [17,21,23]. There are two distinct cognitive interviewing paradigms [22,25]. The “Think-aloud” method requires participants to verbalize their thought process in “real time” as they answer a set of sample survey questions. Retrospective probing or “respondent debriefing” [16] requires participants to complete a set of sample survey questions and immediately respond to detailed probes about the questions.

Because researchers disagree about which method is best [21,23,24], we used a combination [25]. For our initial round of interviews, we used retrospective probes to reduce participant burden and to approximate a real experience of responding to actual survey questions. Once we began to finalize terms, response options and question formats, we asked participants to think-aloud as they responded in order to identify additional problems that might not have arisen with our targeted probes. Interviews were conducted via telephone and lasted between 30 and 60 minutes. Each interview contained between 12 and 27 questions, with additional probes depending on the responses.

To refine components of questions into testable questionnaire items, we completed seven rounds of cognitive interviews, each round being concluded when sufficient agreement or disagreement among participants led to adjustments of both items and probes. Within each round, we made minor revisions to address emergent problems and gain additional insight into the reason for a particular problem (e.g. adding a probe to learn why participant definitions of a particular term were inconsistent). Each round included 4 to 11 interviews. Interview guides were prepared in advance and interviewers were instructed to follow scripted probes carefully in order to facilitate analysis and comparability [26]. Where unanticipated concerns were elicited, interviewers were instructed to add probes as appropriate [23].

Table 1 lists all specific terms tested in any of the seven rounds of interviews, and their sources. Initially, we asked participants to define terms related to expectations and back pain. Terms included in the first interview guide can be found in Table 1: Terms and phrases describing expectations (with the exception of think is likely to occur which was added later); back pain; and back dysfunction and global effects. We asked participants to define new terms throughout the seven rounds of interviews. Later rounds combined terms, contrasted them with one another, and tested complete questions with response sets in varying combinations and orders. Interview guides typically contained 10-12 sample questions and 4-6 probes for each sample question. To create clear questions for our domains of interest, we tested a variety of combinations of terms and phrases describing expectations, back pain, and our response options.

**Table 1:** Comprehensive list of specific terms tested in cognitive interviews

**Broad category**	**Specific terms**	Source for specific terms*
**1. Terms and Phrases Describing Expectations**	Expect	Cambron; Cherkin/Sherman; Coeytaux; Evans; Foster; Haas; Hondras; Qualitative interviews of patients
	Personally expect	Cohen; Linde; Witt
	Realistically expect	Qualitative interviews of patients
	Hope	Qualitative interviews of patients
	hopeful	Foster
	Think	Cohen; Lao
	Feel	Borkovec and Nau (Used by Lewith, Wayne and P. White); Cohen
	Believe	Hondras; Linde; Lao; Vas
	Really think	Devilly and Borkovec used “really feel”
	Successful	Borkovec and Nau; Cohen; Devilly and Borkovec; Haas; Kaptchuk; Hurwitz; Lamb
	Helpful	Cherkin/Sherman; Hondras; Ritenbaugh
	Think is likely to occur	Devilly and Borkovec; Expert reviewer
	Confident	Borkovec and Nau (Used by Lewith, Wayne and P. White); Cambron; Cohen; Haas; Hurwitz; Kaptchuk; Lamb
**2. Domains of Outcome Expectations**		
**Back Pain**	Back condition	Cherkin/Sherman
	Back pain	Back pain *[Cherkin/Sherman; Foster]*
		Low back pain *[Evans; Hondras; Hurwitz; Williams; Witt]*
	Back pain problems	Low back problems *[Hondras]*
**Back Dysfunction and Global Effects**	Limitations due to back pain	*Devilly and Borkovec*
	Impact of back pain on life	Qualitative interviews of patients
**Impact of Back Pain on Specific Areas of Life**	Impact on work	Qualitative interviews of patients
	Impact on social and recreational activities	Qualitative interviews of patients
	Impact on daily activities	Qualitative interviews of patients
	Impact on relationships with family and friends	Qualitative interviews of patients
**Mood, Energy**	Mood	*Moyer;* Qualitative interviews of patients
	Energy	*Cohen*; *Mao;* Qualitative interviews of patients
**Coping**	Coping	Mao
**3. Timeframe**	End of treatment period	*Devilly and Borkovec*
	Short-term outcome	*Hondras (1 month from now)*
	Long-term outcome	*Evans (3 months after)*
	One year from now	*Cherkin/Sherman*
**1. Response Options**		
**Words**	Agreement with item	*Cohen; Mao; Moyer; Vas; White;*
	Word descriptors for each gradation	*Cohen; Cherkin/Sherman; Evans; Foster; Linde ART, Ritenbaugh; Witt; Mao; Lao*
**Numbers**	0 to 10	*Cambron; Coeytaux; Evans; Foster; Hondras*
	1 to 9	*Devilly and Borkovec*
	Percentage	*Devilly and Borkovec; Evans;*
	-5 to-5	Expert reviewer
	1 to 5	*Kaptchuk*
**2. Anchors**		
	Relative descriptors	*Lower anchors:* A little worse [study team]; Much worse *[Cherkin/Sherman; Hondras]*; Very unhelpful *[Ritenbaugh];* Worst pain imaginable *[Cambron]*
		*Upper anchors:* Better than it’s ever been [study team]; Extremely helpful *[Evans; Ritenbaugh]*; Extremely hopeful *[Foster]*; Very effective *[Coeytaux]*
	Absolute descriptors	*Lower anchors:* No pain *[Cambron]*; Not at all effective *[Coeytaux]*; No change/worse [study team]; Not at all helpful *[Williams; Hondras]*; Not at all hopeful *[Foster];* No Improvement *[Witt]*
		*Upper anchors:* Complete Relief *[expert reviewers]*; Completely Better *[Foster]*; Completely Gone *[Cherkin/Sherman; Hondras]*; Cure *[Linde; Witt]*; No pain, pain-free [Cognitive Interviews]

*For citations and additional information see Additional file 1: Appendix A & Additional file 1: Appendix B.

Table 2 lists terms tested in each round of interviews. Underlined terms were ultimately selected for use in our draft questionnaire. Italicized terms were eliminated in that round of testing. We typically tested each term in multiple rounds of interviews.

**Table 2:** Decision-making process for eliminating or modifying questions or terms in the rounds of cognitive interviews

	**Round 1**	**Round 2**	**Round 3**	**Round 4**	**Round 5**	**E.R.**	**Round 6**	**Round 7**
	**(4 ppts)**	**(7 ppts)**	**(4 ppts)**	**(4 ppts)**	**(9 ppts)**		**“Think-Aloud” trial + probes**	**“Think-Aloud” trial + probes**
							**(11 ppts)**	**(4 ppts)**
	2) Expect	2) Realistically expect	2) Hope	2) Hope	2) ***Hope***		2) **Hope**	
	*3) Personally expect*	3) Hope	3) Realistically Expect	3) Realistically Expect	3) Realistically Expect		*3) Think is likely to Occur*	
	4) Realistically expect	*4) Think/feel/believe*	*4) Successful*	*4) How likely (particular outcomes)*				
	5) *Hopeful*		5) Substantially reduce	*5) Substantially reduce VS reduce VS meaningfully*				
	*6) Confident*		VS reduce					
				*reduce*				
	7) Think/Feel/Believe							
	*8) Really think*							
Back pain terms	*1) Back condition*	***1) Back pain***	**1) Back pain**	**1) Back pain**	**1) Back pain**		**1) Back Pain**	**1) Back pain**
	*2) Back pain problems*		*2) Average/current/ worst pain*					
	3) Back pain							
Back dysfunction and global effects terms + outcomes domains	1) Limitations due to back pain	1) Limitations due to back pain	**1) Impact of back pain on life**	**1) Impact of back pain on life**	**1) Impact of back pain on life**		***1) Physical limitations due to back pain***	**1) Impact of back pain on life**
	2) Impact of back pain on life	***2) Impact of back pain on life***	2) Impact on work; social and recreational activities; daily activities; interactions with family & friends	2) Impact on work; social and recreational activities; daily activities; interactions with family & friends	***2) Impact on work; social and recreational activities; daily activities; interactions with family & friends***		**2) Impact of back pain on life**	
	*3) How BP interferes with life*	3) Impact on work; social and recreational activities; daily activities; interactions with family	3) Sleep problems	3) Mood/Irritability	3) Mood/irritability		**3) Impact on work; social and recreational activities; daily activities; interactions with family & friends**	
		4) Sleep problems			***4) Energy***		***4) Back-related sleep problems***	
							***5) Mood***	
							**6) Energy**	
Coping					***1) Coping***		**1) Coping**	
Timeframe		1) ***End of Tx period***		1) End of Tx period 2) 1 year after			**1) End of Tx period 2) One year from now**	***1) One year from now*** (with additional instructions)
Response options	1) words VS numbers	*1) Ppt defined scales*	***1) 0-10***	**1) 0-10**	**1) 0-10**		**1) 0-10**	**1) 0-10**
	2) Percent	*2) Word set (a little worse- better than ever)*	*2) 0-100*					
	3) Likert scale (Strongly agree – strongly disagree)	3) 0-10	*3) Percent*					
		*4) Likert scale (Strongly agree – strongly disagree)*	*4) Negative numbers*					
		*5) Middle Anchors*						
		*6) 1-9*						
Word Anchors		*1) 0 = a little worse*, *10 = better than it’s ever been*	1) 0 = No change, 10 = completely cured/no pain/pain-free	1) 0 = No change, 10 = no back pain	1) 0 = no change, 10 = no back pain		***1) 0= no change/worse, 10 = complete relief***	
			*2) Midpoint Anchor - somewhat*	2) 0 = No change, 10 = back pain does not affect my mood	2) 0 = no change, ***10 = back pain no longer impacts my life***			
				3) 0 = not at all likely, 10 = very likely	***3)*** *Cope VS really cope VS cope well*		***2) 10 = back pain no longer impacts my***: (life) (sleep) (mood/irritability) (energy) (work) (Social and recreational activities) (daily activities) (relationships)	
					*4)* ***NA***			
							***3) 10 = Limitations completely resolved***	
Decisions/Rationale		1) Acupuncture Expectancy Scale eliminated – inconsistent meanings of questions	1) Upper anchor changed to “no back pain”	1) “How likely (specific outcomes)” was changed to questions of “how much change is expected”	1) Tested Cherkin/Sherman question “how helpful…”		1) Eliminated: Think is likely to occur – not as consistent as realistically expect	1) Use order suggested by survey design expert for self-care questions
		2) “Limitations due to back pain” kept for later, not tested in all rounds due to clarity of concept	2) Ppts prefer same scale throughout questionnaire		2) Work on Energy as a domain – test “energy level”			

*Key.

Italicized text = eliminated.

Bold italicized text **=** selected for use in final questionnaire.

E.R. = Expert Review (prior to round 6).

Additional tables provide quotations illustrating findings reported in the main text. For most findings, one quotation was selected that best represented overall findings. In situations where we found inconsistent definitions, multiple quotes are often included to illustrate conflicting views.

The following situations raised concerns about specific questionnaire items: (a) inconsistent responses among participants; (b) responses that differed from our a priori expectations; (c) participants reluctant to answer questions or confused about how to do so; or (d) terms or phrases that our participants found to be ambiguous. We continued to test items in cognitive interviews until a satisfactory level of category saturation [27] was reached. Some items were eliminated quickly, but others required multiple adjustments and rounds of questioning before they were either eliminated or included in the final questionnaire. No items were accepted without being tested in at least two rounds of questioning and in varying order within the questionnaire.

Cognitive interviewing is not intended to yield the best possible question, but rather to provide information to facilitate the design of clear and logical questions [21]. After five rounds of interviews, we sought feedback on a draft questionnaire from seven colleagues with expertise in survey design, CAM research, research on chronic pain, and/or patient expectations. After receiving their feedback, we modified the questionnaire and conducted two additional rounds of cognitive interviews (Table 2). We then drafted a cognitively-informed questionnaire that we will test psychometrically.

### Interviewers and training

All four cognitive interviewers had considerable experience in qualitative interviewing and were familiar with the goals of the study. CR had previous experience conducting and analyzing cognitive interviews for questionnaire development [17] and designed and conducted a cognitive interviewer training. Digital recordings were quickly transcribed for immediate review by cognitive interview team members. Rapid turn-around on transcription provided opportunities for assuring comparable interview approach and probing. Weekly teleconference team meetings were the venue for assessing the results of that week’s interviews, modifying the interview process as needed in real time, and making prompt decisions for next steps. The high level of interaction among interviewers and investigators assured comparability across interviewers and sites.

### Participant recruitment

We recruited a convenience sample of 39 adults (22 from Arizona, 17 from Washington State) with chronic back pain through recommendations from CAM providers, fliers posted in the community, word of mouth, and internet advertising. Eligibility criteria included: ages 20 to 64; back pain lasting at least 3 months; and no experience with at least one of the four therapies of interest (acupuncture, massage, chiropractic, yoga classes). Participants were not required to be starting a new therapy, but only to express interest in trying one of the four target therapies and to be naïve to that therapy. These inclusion criteria were useful for finding participants who would resemble patients willing to enroll in a clinical trial of CAM. Both the Group Health and University of Arizona Institutional Review Boards determined that these cognitive interviews were “not Human Subjects Research” because the questions were non-sensitive, hypothetical and used for questionnaire development and therefore did not require that we provide formal informed consent. However, prospective participants who responded were provided with a brief description of the study and screened for eligibility. Eligible participants were provided with complete study description, an explanation of cognitive interviews, and information about privacy and protection of the data collected.

### Data analysis

Cognitive interviews were audio-recorded and transcribed verbatim. Interview transcripts were later coded using qualitative data analysis software (ATLAS.ti, Version 6.0 [28]) by EE, an interviewer experienced in coding and analyzing qualitative data. Coding was helpful for confirming or refuting initial impressions and for organizing the data for presentation to the study team.

Because interviewers had been instructed to use scripted probes and improvised only to elicit additional data, coding was simple and typically aligned with pre-determined domains and areas of interest. 86 codes covered the terms tested (e.g. terms about expectation, response anchors), outcome domains (e.g. back pain, sleep, mood, energy, coping), timeframe and response options (e.g. 0-10 scale, percentage scale, participant defined scale), and comments pertaining to a specific question (e.g. questions originally used by DC and KJS or specific questions created based on participant comments).

## Results

### Components of the questions

#### Terms and phrases describing expectations

We tested numerous terms to see how useful they were in eliciting meaningful responses related to expectancy (Table 1). In the first round of interviews, we asked participants to define each term and tell us which they preferred. In later rounds of interviews we continued to clarify which terms were most consistently defined and most meaningful to participants. Table 3 provides relevant quotes for each term.

**Table 3:** Components of the questions: illustrative quotations for terms and phrases describing expectations

**Term or phrase**		**Quote**
3.1	Expect	Expect, is that like another word for hope? Because that’s how I’m using it. Do you know what I mean? Because I’m sort of like, I don’t know what to expect, but I sure have a lot of hope. So I think that’s, if this is important for you, I don’t know, but that’s how I’m using the word expect. AZ-518
3.2	Realistically Expect	If you were to ask me what do I expect, do I talk about what I expect realistically? And then I might talk about what I hope for. But if you were to ask me, what do I realistically expect, if I just focused on what is realistic, what is a realistic outcome. SEA-316
3.3	Hope	Well so whenever I try a new treatment there’s always a hope in the back of my mind that I’m hoping that I’ll be completely free of the back pain and feel completely well. That hasn’t been my true experience, but there’s always that hope, you know? AZ-427
3.4	Think is likely to occur	Likely to occur to me, is taking it a little bit more distance and a little bit more objectively. Like, based on my research, it’s likely that I’ll have an 8, but what do I realistically expect might bring in some more of my pessimism about what might happen for me.” SEA-803
		“I’d probably say expect [is more meaningful] because likely, I don’t know. Likely means, sort of means to me more like a guess. But expect is more like your opinion.” AZ-817
3.5	Feel	“Feel” is more vague. Like it’s almost more like it’s asking you what your intuition is about treatment. More like a gut reaction as opposed to thinking about it and like, processing all of the information that you have. SEA-316
3.6	Think AND Believe	I don’t hear a different in “believe” versus “think”. I think you have to be pretty sophisticated to register a difference on that and so I think in using either verb, it’s the same question to me. SEA-531
3.7	Helpful	Helpful, I guess means steps to recovery, not an instant fix, but something that you work at. AZ-529
3.8	Successful	Either 95 to 100% pain free. But I would also say it would be successful if it would be a temporary relief of pain, too. In other words, there’d be a temporary period of relief as opposed to a more permanent period of relief. SEA-501
3.9	Confident	I think confident is basically the same thing as hope, but not as positive. AZ-219
		Well to me it’s contrary to hopeful. I keep going back to that because, to me, wishful is a very soft, gentle word that is not clear. And confident is, I’m confident. Because it’s been explained to me. AZ-313
3.10	Hopeful	Hopeful is like, well I just hope something good happens. I’m hoping something good will happen. AZ-313

Originally, we anticipated the term expect, which participants consistently defined as what people think will happen as a result of treatment, would be the best way to ask about this belief. However, after several rounds of cognitive testing, we learned that *expect* was not actually used consistently when participants responded to sample questions containing the term (Table 3). Some participants answered based on their hopes while others considered both their hopes and expectations. Participants initially defined the terms *expect* and *realistically expect* similarly. However when probed, they described *realistically expect* as more narrowly focused on what one really thinks will happen without consideration of one’s hopes (Table 3). Some participants said the word “realistically” made them think about what they really thought would occur.

In cognitive interviews, participants defined *hope* as what they wished for or wanted to occur at the highest levels of aspiration, unconstrained by reality, prior knowledge or experience (Table 3). Some participants said *hope* includes emotional aspects of what is expected. By contrast, the term *expect* is more realistic. Based on these findings, we chose to pair questions about *hope* with questions about *realistically expect* in our questionnaire in order to tease apart blind hopes from more realistic beliefs.

One of our expert reviewers recommended we use the phrase *think is likely to occur* (Table 3) instead of *realistically expect*. Our participants defined these two phrases similarly. When we compared numerical responses to both survey items, however, we found that participants typically used the same numerical rating for *think is likely to occur* as they used for the question immediately preceding it (e.g. a high number if it followed a question containing *hope*; a low number if it followed a question containing *realistically expect*). In contrast, responses to *realistically expect* and *hope* were consistently divided and did not seem to fluctuate with question order. We therefore eliminated *think is likely to occur* from further consideration.

### Other terms tested

*Think*, *feel*, and *believe* (Table 3) were tested as ways to ask participants about their expectations for treatment outcome (e.g. How successful do you feel the treatment will be in relieving your back pain?). We eliminated them because, compared to *realistically expect* and *hope*, they did not elicit consistent responses about participant expectations and anticipated outcomes.

Participants found *personally expect* redundant, since they assumed all questions were about their beliefs. We eliminated *really think* because participants described feeling as though this questioned their honesty, which this had a negative connotation.

We eliminated *helpful*, *successful*, *confident*, and *hopeful* because participants defined them inconsistently or we found them less useful in capturing expectations (Table 3). *Helpful* was defined by some as steps toward relieving pain, and by others as related to consequences of improvement in pain. *Successful* was defined by some participants as a marked improvement, while others thought this meant that the treatment worked as expected. *Success* seemed focused on the end of the treatment period and did not imply long-term improvement. *Confident* was not consistently defined (Table 3). *Hopeful* was distinct from *hope* (described above) and was consistently defined as a broadly wishful or optimistic approach. *Hopeful* is less goal-oriented than *hope* and we therefore chose to use the term *hope* in our draft questionnaire due to its association with more specific outcomes.

### Domains of outcome expectations

#### Back pain

In order to assess the usefulness and meaningfulness of different terms used to describe back pain, we cognitively tested several terms or phrases used by past researchers, including: *back condition*; *back pain;* and *back pain problems* (Table 4). In the first round of interviews, participants were asked to describe the meaning of each term or phrase independently, and then to compare them to one another.

**Table 4:** Components of the questions: illustrative quotations for domains of outcome expectations

***Outcome domain***		***Quotes***
*4.1 Back Pain*		
4.1.1	Back Pain	I would say [back pain]’s more specific to the pain itself, like where it hurts, how it hurts, how often it hurts, the characteristics of the pain. SEA-316
4.1.2	Back Condition	I think back condition could be things that may not be physically felt by the person. Even you know, spinal cord related problems. AZ-219
		[Back condition] is pretty broad because it can entail the whole back. From the cervical area all the way down to the sciatic area. AZ-312
4.1.3	Back Pain Problems	Definitely pain, discomfort. Let’s see, I know that some sort of alignment of the spine affects it too. Pretty sure that’s more medically related. Yeah, I would probably just say pain. AZ-219
		Problems, an inability to move, do things, bend over, pick things up, hold grandbabies, pick them up, get up and down off the floor. Things like that. AZ-312
4.1.4	Back pain: average/current/worst	I would say probably more like average pain. But also thinking about those times when I’m particularly uncomfortable…Average was where I went immediately, sort of a global average pain level. AZ-511
*4.2 Back Dysfunction and Global Effects*		
4.2.1	Limitations due to back pain	Limitations due to back pain makes me think more so of like specific physical activity, general activity limitations, whereas impact is more broad than something like medical, social, physical, like a broader spectrum of effects. SEA-316
		What I’m able to do. What limitations do you think of? Not sitting for a long time. In my case, not being able to do certain poses in yoga. And in my case it’s mainly sitting. I’m not supposed to sit for a long time. AZ-312
		Limitations to me means not being able to do specific tasks, whether it’s cleaning the house, bending over, doing yard work, lifting the recyclables. You know, opening a bottle… just being able to do day to day things like you used to do. AZ-313
4.2.2	How back pain interferes with life	Kind of like limitations due to back pain. How pain interferes with your life, how the pain affects your life… how back pain interferes is like more active, so it makes me think more of activities. SEA-316
4.2.3	Impact of back pain on life	The impact, yeah. I think it’s broader, to me, than going right into the specific limitations… More emotional and general life kind of things, than specifically… limitations still to me, deals with something physical. Where impact is I think broader. That opens the door to other things. AZ-313
		Activity level, everything, mood, like all levels of my mental state, ‘cause when you’re in pain, it’s like, to be crabby all the time or to be in pain, it’s, yeah, and then you can’t focus at work, or you know. It affects everything. SEA-515
*4.3 Impact of Back Pain on Specific Areas of Life*		
4.3.1	*Comments about how general question about impact of back pain on life was interpreted*	Well I don’t think I was [thinking about emotional impact] until you asked me about it. Until you break it down into all those little pieces. It’s important to specify all those questions. Because I think it makes you think about it differently, a little bit. AZ-416
4.3.2	Impact on work	To me, it’s a different question because although I may have back pain at work I will exert more through the back pain and work through it, you know what I mean?… If you’re having a lot of pain at home, you can opt, “Okay, this day, I won’t go gardening out on the rockery” But I might tomorrow, when I feel better. Something like that. But at work, if you have to do the thing today, you’re gonna do it. SEA-501
4.3.3	Impact on social and recreational activities	Well, to me [social and recreational activities] sounds like, more about the outside, you know, gardening. Other things that I get enjoyment of out. But when you say life, I think of all aspects of my life. Or in my case, my work. AZ-416
4.3.4	Impact on daily activities	But also the overall quality of life, or however the first one is worded really averages things out, and daily activities I’m thinking about the times that I’m active during the day. Not so much nighttime activities like sleeping. AZ-508
4.3.5	Impact on relationships with family and friends	Yeah, because I was thinking impact on my life I was thinking more generally about what I’m able to do. When I started to think about impact on my family I was thinking if I was in less pain, if I had anything better then my family would be much happier. Because I’m also crabbier than I would be if I wasn’t in pain. AZ-427
*4.4 Sleep, Mood & Energy*		
4.4.1	Back-related sleep problems	I think I chose slightly higher than what I expected to get from the back. So if my back was a 2 then I chose slightly higher because I feel like any improvement in the pain might make even more improvement in the sleep. AZ-427
4.4.2	Mood	Not having back pain would allow me to not be focusing on that pain, and not getting agitated as easily… I think the treatment itself would also provide a space for me to relax mentally and physically. Just taking the time to take care of myself, instead of just trudging through the day with the pain. AZ-508
4.4.3	Energy	Yes, anytime we do anything for my back I want… back pain is an energy suck, and anything you’re doing, I think people are gonna be hopeful that back pain will end up having less energy drain. SEA-531
*4.5 Coping*		
4.5.1	Coping	Coping to me just means how well I’m dealing with the pain. Can I operate even though there is pain? AZ-530
4.5.2	Coping VS Self-care	When you say “self-care” I think, okay, I need to do things, remembering to stretch and doing meditation or maybe taking ibuprofen at the end of the day or something, that to me, is self-care. Coping is just kind of psychologically like, “Can I get through the day? Can I do this activity that I need to do?” SEA-MP-601

Of the three terms describing pain, participants understood *back pain* most clearly. They consistently defined *back pain* as the physical experience of pain, while *back condition* was described as a more anatomical or diagnostic term that some participants did not believe was relevant to their symptom experience. *Back pain problems* was slightly more ambiguous, referring to the physical problems associated with back pain according to some respondents, and to how back pain interferes with life for others.

#### Back pain: average/current/worst

We also tested the value of specifically asking about *average pain*, *worst pain*, and *current pain* (Table 4)*.* When asking participants whether they had described their *average pain*, *current pain* or *worst pain*, most (6 of 7) said they had described average pain. While they preferred *average pain* to the other terms, they preferred to think about areas of highest importance for them, or to answer based on a broader average. Participants had trouble compartmentalizing pain into these categories. They reported it was easier to respond if they could consider both the severity of acute episodes and the constant presence of low or mid-level pain. They did not believe that the term *average pain* captured these considerations. Based on these findings, we elected to use *back pain* in our draft questionnaire without additional qualification.

#### Back dysfunction and global effects

To evaluate appropriate terms for describing “back dysfunction” [29] and “global improvement” [30] we tested: *limitations due to back pain; impact of back pain on your life*; and *how back pain interferes with your life* (Table 4). Participants said both *limitations due to back pain* and *how back pain interferes with your life* referred specifically to physical limitations. *Limitations* and *interference* were consistently understood to mean physical inability to complete specific tasks. However, the *impact of back pain* on participants’ lives was consistently defined more broadly. It included less tangible aspects of life such as irritability, mood, energy, sleep, and “lightness of being”. All of these domains were explicitly *not* considered to be “interference” and respondents did not include them when answering sample questions about interference. For example, some participants with back pain who continued to *do* their normal activities reported no interference. However, the same participants reported that the back pain impacted them by increasing irritability, decreasing their enjoyment or “ease and joy” in completing tasks and therefore had a significant impact on their life.

#### Impacts of back pain on specific areas of life

We included some questions about expectations related to specific areas of life that back pain could affect but that participants might not have routinely considered as part of general questions about expectations of treatment (Table 4).

Specifically, we tested questions about: (a) *impact of back pain on work, including housework*; (b*) impact of back pain on social and recreational activities*; (c) *impact of back pain on daily activities*; and (e) *impact of back pain on relationships with family and friends. Impact of back pain on work* was important to include because some participants felt was the most important area of impact while others said they tended to power through pain while at work in ways they could not in other circumstances. Many participants had eliminated sports or other leisure activities from their lives, and did not always consider these activities when responding to general questions about impact of back pain on life. *Daily activities* were defined by participants as a focus on active times or things that needed attention on a daily basis, rather than on an impact in general. *Relationships with family and friends* was the domain in which many participants said they were most likely to be impacted by their pain. Families were impacted by negative moods stemming from pain, although this was rarely mentioned in responses to more general questions.

#### Sleep, mood, and energy

Poor sleep is a known consequence of back pain for many patients [31]. We used the phrase *back-related sleep problems* since some participants said they reported sleep problems unrelated to pain (Table 4).

We decided to include questions about *mood* and *energy*[30] as some participants reported difficulties in these areas as consequences of back pain (Table 4) [32]. Participants’ found these terms straightforward. For these two domains, participants reported they might expect treatment to have benefits not directly correlated to reduction in pain.

#### Coping

We included coping as a domain of experience because improvement in coping was distinct from improvement in pain (Table 4). Our participants defined coping as the mental ability to carry on in spite of pain. Participants distinguished between *coping* and *self-care* very consistently in our interviews. While *coping* was the ability to handle pain, *self-care* referred to one’s ability to employ various strategies aimed at decreasing or preventing pain.

### Timeframe for improvement

We wanted to assess participants’ short-term and long-term expectations. We tried several terms, including *end of the treatment period* and *one year from now*. Participants thought the term *at the end of the treatment period* typically referred to a period between 6 weeks and 6 months, with most (7 of 10) thinking this was 10 weeks to 3 months.

Given the high degree of agreement, we left the end of the treatment period open, though it could be clarified for future studies if needed. To ask about expectations of long-term outcomes, we tested multiple versions of a question about expectations for *one year from now*. Simply asking what participants expected *one year from now* was confusing because some participants could not predict long-term outcomes in the absence of knowing the short-term results. Other participants did not know whether they should include other treatments they might do during the year. We therefore explained that participants should include the current course of treatment they are seeking, plus any self-care or other health care they anticipated completing during that time period. By including this domain in our questionnaire, we can test whether participants expect their treatments would have short term benefits for back pain or would have more lasting benefits.

### Components of the responses

#### Response options

Overall, we found participants preferred response options that were consistent throughout the questionnaire, that numerical options elicited more consistently meaningful responses than word sets, and that numerical rating scales were the most intuitive for participants because they were typically asked about pain on a similar scale in health care settings.

### Word sets

We tested several types of word sets as potential response options (Table 5). Although some participants said they preferred word options, many reported difficulty in remembering the words during their telephone interview. In addition, the meanings of some words differed among participants, and having words define each option made the scales appear non-linear to participants. Finally, participants found it easier to be asked questions using the same scale throughout the interview.

**Table 5:** Illustrative quotations for response options

***Response option***		***Quote***
*5.1 Word Sets*		
5.1.1	*Issue:* Participant difficulty recalling words	*Participant attempting to recall response choices: “*Disagree strongly, disagree slightly, agree, agree moderately agree slightly, don’t have a clue. Don’t know what you just said. I would need to have these written down or write them down myself to look at otherwise I can’t remember and I spend more time trying to think memorize the answer choices but forget the question.” SEA-416
5.1.2	*Issues:* Meanings of words inconsistent among participants; leads to non-linear scale	“When you started giving options, I sort of, in my mind, pictured the one going up to ten, so I think your first option was a little worse, if that was the scale, that would be the one. And as it went up, “back feels better than before”, from the treatment, I figured that as the ten and you sort of mentioned, the option after that, it sort of felt like it went from one to ten and then sort of went backwards again, it was a natural order in my mind, so, what was the option after “back pain”?” SEA-412
*5.2 Agree-Disagree Likert Scale (adapted from Mao*[35,36]*)*		
5.2.1	Item 1: “My pain will improve a lot”	“I just felt confused, like do I say I agree with the statement? Do I have to disagree with the statement? I just felt like ugh. I feel like depending on how a question was worded, especially if it was negative I feel like I could easily have said the opposite of what I meant. Just by being confused.” AZ-411
	*Respondents confused about how to convey their expectations on this scale; not in agreement about appropriate response*	

		Scenario (1): If you only thought your pain would improve a little, what response would you choose?
		- I would probably say disagree moderately… I’m disagreeing moderately that my pain will improve a lot, because I’m still trying to keep an open mind but I am sort of feeling like I’ve tried so many other things that I’m not really sure if it’s going to help that much. SEA-410
		- Agree slightly. I would endorse it slightly with “A little bit” toward a smaller degree. Then moderate would be sort of, I think the next step up, but not “agree with strongly” which would be a lot of improvement. I think “more slightly” is the lowest degree of positive, if that makes sense. SEA-412
5.2.2	Concerns with interpretation of Item 2: “I will be better able to cope with my pain”	“If I’m not coping anymore do I say strongly disagree or I strongly agree? The question doesn’t make sense anymore with an agree, disagree kind of response.” AZ-411
5.2.3	Item 4: “My energy will improve” *Responses to scenarios lacked consistency among respondents*	Scenario (1): If you thought your energy would increase just a little, what would your response be?
		- I slightly agree? When I was trying to say, or moderately agree is what I would respond to try to say it increases a little bit. AZ-411
		- I would probably say agree moderately SEA-410
		- Neither agree nor disagree SEA-416
		- Agree slightly. SEA-412
		Scenario (2): If you thought your energy level was fine now, how would you answer that?
		- Huh, my energy level is fine, I’d probably say I don’t agree to that statement? AZ-411
		- I think I’d still slightly agree. SEA-416
		- If I felt my energy level was fine, I would say “neither agree nor disagree.” SEA-412
*5.3 Numerical Response Options*		
5.3.1	0-10 Scale	“There was a movie out once where they called a girl a “10” because she was very good looking. So zero to ten is kind of, 10 has always been “the best” and zero is “no good”. And that’s the same scale they use for pain, zero to ten.” AZ-813
		“I think the one to ten scale is kind of the easiest thing for people to relate to, I don’t think people relate very well to using percentages or negative numbers, I think they would just find that confusing. One to 100 seems a little bit arbitrarily more detailed than you need it to be. I mean is somebody gonna choose a 37.4 out of 100?” SEA-430
5.3.2	0-100 Scale	“I like zero to ten better… It was just like, oh, there’s so many more numbers to choose from. Like 44 or 88… the question is too general for that sort of precision but there was space for that sort of precision so I was like, oh…” AZ-427
*5.4 Anchors*		
5.4.1	Concrete upper anchor, support	What does “extreme improvement” mean to you? It means total relief. Total relief would be the same thing. Total relief would be better, but extreme improvement is still okay. it still gives me a good idea of what you’re asking. AZ-816
		I think it was easy, but I’m actually thinking a better word would be total improvement rather than extreme improvement. ‘Cause zero’s no improvement, so ten would be total improvement. It would be a better choice.’ SEA-803
5.4.2	Lower Anchor: No Change/Worse	If there is no change or getting worse it’s going to be zero. If there is like complete relief of pain that’s going to be ten. So it makes sense. AZ-529
5.4.3	Upper Anchor: Complete Relief	I think complete relief speaks more clearly. Yeah. Because it’s referring to the pain. But then again completely resolved does put it at, it’s the main problem in your life too. Which it does create depression and a huge change in everything in your life. So I can also relate to that. AZ-831
		I would say completely resolved makes more sense because, well, given my own thoughts about it, because completely resolved to me, means it’s not a problem anymore, don’t worry about it, complete relief could be, it’s good for now, but it might come back later. And I think that resolved is a more solid result. SEA-803
		I kind of like relief better, ‘cause it really describes more, I think, what people are after with back pain.’ SEA-831
5.4.4	Reliance on midpoint anchor	I think in some ways when you do that slightly moderately part because the variation is sometimes so little and you have to twist your head around to see how your answer fits with those words-it’s hard to pick. I always find myself doing these things to one side of the middle. SEA-416
5.4.5	Discrepancy in midpoint anchor word meaning	I thought there was going to- how much improvement- to me the scale, when it was 1 to 10 before I had put two or three as somewhat, but then you changed that to 5 so I chose 5. SO YOU CHOSE 5 BECAUSE I SAID 5 IS SOMEWHAT SUCCESSFUL? Yeah, on a zero to ten I thought of 5 as like 50% improvement, so to me somewhat successful is a bit less than that. Somewhat successful to me is like 20 to 30%. 50% improvement is more noticeable. AZ-427

We tested Likert scales reflecting degree of endorsement. Responses included: *strongly disagree*; *moderately disagree*; *a little disagree*; *neither agree nor disagree*; *a little agree*; *moderately agree*; *strongly agree* (used by [33,34] and more recently by Younger et al. [15]. To test this set of responses, we adapted four items from Mao’s validated “Acupuncture Expectancy Scale” expectations of acupuncture [35,36] (Mao)^a^: 1) “my pain will improve a lot”; 2) “I will be better able to cope with my pain”; 3) “my pain will go away”; and 4) “my energy level will improve”. We discovered that participants were confused about the use of these response options (Table 5 for examples on items 1, 2 and 4). Respondents reported different interpretations of these questions when telling us how they answered these questions. They had trouble reconciling the value judgment in the stem (*improve, get better*) with a value judgment in the response category (*moderately, strongly*). Furthermore, when given hypothetical scenarios respondents did not consistently select the same response category.

For example, responses to item 2 (ability to cope) revealed general issues with agree/disagree responses as demonstrated by responses to two scenarios. Scenario (1): Participants were asked, “If you thought your pain would be completely gone, how would you answer this question?” All three participants said they would strongly agree. Scenario (2): participants were asked “If you thought your pain would stay the same but you would have new tools for dealing with it, how would you answer this question?” In response, all three participants also said they would strongly agree. In this example, two different situations elicited the same response. These responses would be hard to interpret if researchers could not distinguish whether a response of “strongly agree” meant a participant expected little relief from pain, but better coping, or significant relief from pain.

### Numerical response options

When asked to define their own scales in response to questions about outcome expectations, all participants selected a numerical scale with the smallest number (0 or 1) representing no change and the largest (5 or 10) representing the best possible outcome (Table 5). No participants preferred a 0-100 scale because the large number of choices was too great for general questions about expectations. Some participants conflated percentage with a 100-point scale. Ultimately, we eliminated percentages in order to use a consistent format for all response options. The 0-10 scale was preferred by most participants, being described as more intuitive, more familiar, and allowing them to communicate their anticipated improvement with a reasonable amount of precision.

### Anchors

After determining that participants preferred a 0 to 10 scale, we tested several possible phrases for the upper and lower anchors as well as a possible midpoint (Table 5). Participants preferred word anchors that described absolute amounts of improvement (e.g., “no change” or “complete relief”) as opposed to relative improvement (e.g., “worst pain imaginable”). Our anchors were tested and modified over several rounds of interviews to determine the best fit for each question and to elicit the most consistent responses.

We eliminated midpoint anchors because participants often did not agree on the definition of the word and relied too heavily on the midpoint anchor rather than choosing an answer based on their own expectations. One participant changed her answer when a midpoint was used because she perceived that definition was below her idea of the midpoint. For most of our questions, the lower anchor “no change/worse” was clearly understood and elicited the most consistent and meaningful responses. Upper anchors were tailored to specific questions. For *back pain* related questions, the upper anchor “complete relief” was found to be most clear and consistent. For questions related to *limitations due to back pain*, we chose “limitations completely resolved” and for questions related to *impact of back pain on life* we chose “back pain no longer impacts my life”. When asking about specific items, we adjusted this anchor to “back pain no longer impacts my: sleep, energy, work, etc”. For our coping question (#8 in draft questionnaire, Additional file 1: Appendix C), “no improvement” to “extreme improvement” was found to be the most meaningful anchor pair after testing several questions and anchor sets.^b^

### Overall structure of questions

In the course of our cognitive interviews, we found that respondents had difficulty specifying the *likelihood* of a specific outcome (e.g., cure, substantial improvement, better coping). Rather, they wanted to tell us how much improvement they *expected*. For example, one participant responded to the item, “my pain will improve a lot” by saying that she was “making in [her] mind [her] own rating system which these [response options] represent” (SEA-416) to allow her to communicate how much improvement she expected. Rather than use the response options as intended, she described changing the scale in a way that enabled her to convey the meaning she intended to convey. Another participant, when asked how *likely* the treatment was to *substantially reduce back pain*, said “I know that the question was slightly different [from how much improvement do you expect], but … I think I kind of was answering it the same way. … In the back of my mind I was thinking about what I expected in terms of relief from pain [*rather than speculating on the likelihood of the statement provided*].” (AZ-511).

This pattern of participant comments and responses led us to think that the ideal question structure would be: “how much change do you [realistically expect] in your [outcome of interest, e.g. back pain]?” where the bracketed ([ ]) terms could vary. The best response options associated with these questions is a scale of zero to ten where zero is “no change or worse” and ten is “[customized version of relief of the problem, e.g. complete relief]”. For example: “On a scale of zero to ten, where zero is no change or worse and ten is complete relief, how much change do you realistically expect in your back pain?”

## Discussion

We have presented findings from the cognitive interviewing phase of our study aimed at creating a questionnaire for more accurately measuring patient expectations of CAM therapies. Through these interviews, we gained insight into a number of key problems that might arise with existing questions now used to assess patient expectations in clinical trials. These key difficulties would not have emerged without cognitive interviewing. Key findings from these interviews included: (1) participants used the term *expect* in an ambiguous way, encompassing both hopes and realistic expectations; (2) participants had difficulty in determining their “average” or “current” pain, in contrast to their lack of difficulty thinking about their back pain in more general terms, i.e. “your back pain”; (3) participants reported a difference between *physical limitations* and *impact of back pain on life* and tended not to report important aspects of impact on life when asked only about limitations; (4) participants did not consider some specific areas of impact unless asked directly (e.g. leisure activities); (5) participants identified sleep, mood, and energy as important areas of potential improvement, independent of pain; (6) participants were confused about the meaning of “improvement in coping” (wondering whether “better coping” did or did not imply a decrease in pain); (7) participants had trouble assessing long-term expectations, which were contingent on a number of lifestyle and other factors; (8) participants adapted some categories of response options to convey what they wanted to communicate with researchers rather than providing the information the question was intended to elicit; (9) participants had problems with response options based on word (as opposed to numeric) descriptors both in relation to ability to recall them (in telephone interviews) and to varying interpretations of word meanings; (10) participants preferred anchors that specified absolute amounts of improvement; and (11) participants desired to communicate how much improvement they expected rather than their speculations on the likelihood of specific outcomes. In order to elicit meaningful, consistent responses, questions need to be framed to take these issues into account.

Other researchers have distinguished between “probability expectations” (what participants think is likely to happen) and “value expectations” (what participants want, feel they need, feel they are due, or what they hope for) [11,12]. While we did not base our cognitive interview questions on these distinctions, they are useful for explaining the reasoning behind our inclusion of both *hope* and *realistically expect* in our questionnaire items. Since participants seemed compelled to disclose their hopes along with any admission of “realistic” expectations, it was important to ask about both, even if we were primarily interested in the latter. In interviews with individuals beginning a new CAM therapy in an earlier phase of this study, we found that participants often described both their hopes and what they really thought would happen when they were asked what they expected from treatment. Eaves et al. [37] provide a framework for distinguishing between different kinds of hope and show how hope is tightly bound to individuals’ assessments of their expectations, experiences, and outcomes of a treatment.

Our findings indicate that cognitive interviews serve an important purpose in survey design. Many questions initially favored by members of the research team and suggested by other experts turned out to present unforeseen interpretation challenges to participants. Our findings are an important reminder to researchers intending to create new questions: researchers’ understandings and usages of words and phrases may differ from those of study participants. Cognitive interviews are instrumental for learning how participants will interpret questions and what meanings participants are attempting to convey with their responses. The latter issue is important not only to ensure validity and consistency of responses, but also to ensure that questions capture what is most important to participants.

Our findings have implications for selecting outcome measures for clinical trials evaluating treatments for back pain, especially treatments utilizing novel therapies. They suggest that some standard questions used in back pain research to measure expectations are more challenging for patients to answer and may need to be reconsidered. If corroborated in other patient populations, our findings strongly suggest that some question structures (such as endorsement of the likelihood of particular outcomes) are problematic regardless of whether they have been validated.

Cognitive interviews add unique insights that complement other tools we have for survey development such as open-ended qualitative interviewing and conceptual analysis or quantitative psychometric analysis of survey responses. Cognitive interviews allow for the blending of quantitative and pattern analysis of numerical rating as well as the qualitative aspects of participants’ discussions of the meaning of those ratings. For example, we found that asking participants what they thought was “likely to occur” as a result of treatment was problematic. The weakness of this term in eliciting the desired information was revealed only when the numerical responses chosen by participants were compared to their descriptions of their intended response and the changes in descriptions and responses when the order of questions was adjusted. Although some researchers may have little experience with this technique and it seems to be infrequently reported, Cognitive Interviewing has been recommended by the Food and Drug Administration (FDA) as part of the development of Patient Reported Outcome (PRO) measures as it is key in ensuring understanding and completeness of conceptual content of questions [18].

In addition to our specific findings, we offer the following insights to researchers planning to conduct cognitive interviews: (1) employing both retrospective probing and “think-aloud” cognitive interviews may elicit a range of issues and help to clarify questions; (2) attention to both participants’ descriptions of their response choices and intended meaning as well as to numerical patterns in their responses is key to understanding data after it has been collected; and (3) gaining an understanding of what participants’ want researchers to learn, and creating questions that elicit that information, leads to more consistent and meaningful responses.

### Limitations

While we anticipate that our results are broadly applicable among English-speaking North Americans, due to cultural differences in health care and social discourse on pain these results should probably be confirmed in local pilot studies prior to use in other English-speaking countries. Translation into other languages will necessitate additional work using standard protocols for survey translation.

## Conclusions

Our goal of creating a standard questionnaire to measure expectations at the beginning of clinical trials is intended to contribute to understanding the complex relationships of patient expectations and treatment outcomes. The cognitive interview phase of our project has added a level of clarity to our questions that we hope will assist our final questionnaire in eliciting information that is not only meaningful to research participants, but also better suited to provide clear data on the links (if, in fact, there are any) between participant expectations and treatment outcomes.

The final steps in our research include: (1) psychometric evaluation of our draft questionnaire in both clinical and clinical trial populations and (2) analysis of outcomes data collected in clinical trials administering the questionnaire. Once these additional steps have been completed, we will provide the research community with a questionnaire on expectations developed specifically for use in CAM settings that they may choose to use in their own future research.

## Endnotes

^a^Mao’s original scale contains the following responses: “not at all agree; a little agree; moderately agree; mostly agree; completely agree”, the study team elected to use “strongly disagree; moderately disagree; slightly disagree; neither agree nor disagree; slightly agree; moderately agree; strongly agree”.

^b^We recognize that “extreme improvement” does not fit with our finding that the upper anchor should communicate an absolute amount of improvement. However, as described in relation to Mao’s coping question [35] above, improvement in coping is not necessarily correlated to improvements in pain and is therefore problematic. We chose to include this domain, but found that the word anchors that best described this question differed from those in other questions.

## Abbreviations

CAM: Complementary and alternative medicine; FDA: Food and drug administration; PRO: Patient reported outcome.

## Competing interests

The authors declare they have no competing interests.

## Authors’ contributions

KJS is the Principal Investigator; she conceived of and designed the overall study, participated in a review of the literature and contacting CAM researchers for copies of the questions on expectancy they had used in prior studies, participated in creating and revising interview guides, analyzing data, compiling a draft questionnaire, contacting content experts to review the draft questionnaire, and drafting and revising the manuscript. ERE participated in creating and revising the interview guides, interviewing participants, coding and analyzing data, and drafting and revising the manuscript. CR participated in design and conceptualization of the overall study and the cognitive interviewing phase of the study, creating and revising interview guides, training cognitive interviewers, coding and analyzing data, compiling a draft questionnaire, and drafting and revising the manuscript. CH participated in creating and revising interview guides, interviewing participants, compiling a draft questionnaire, and provided important intellectual insight and revision of the manuscript. DC participated in the design of the overall study, contributed important intellectual insight and revisions to the manuscript. JT participated in the design of the overall study, reviewing the literature, contacting content experts to review the draft questionnaire, compiling a draft questionnaire, and contributed important intellectual insight and revisions to the manuscript. All authors read and approved the final manuscript.

## Pre-publication history

The pre-publication history for this paper can be accessed here:

http://www.biomedcentral.com/1472-6882/14/39/prepub

## Supplementary Material

Additional file 1 **Appendix A.** List of researchers responding to requests for contributions of expectancy-related questions used in their clinical trials. **Appendix B.** Representative citations for authors contributing questionnaire items or with published questionnaires of interest. **Appendix C.** Draft EXPECT Questionnaire (after Cognitive Testing). Click here for file
