# Supplementary material for: Cognitive interviews guide design of a new CAM patient expectations questionnaire
Source: BMC Complement Altern Med. 2014 Jan 25;14:39. doi: 10.1186/1472-6882-14-39 (PMC3906834; doi:10.1186/1472-6882-14-39)
Supplement: Additional file 1 — Appendix A. List of researchers responding to requests for contributions of expectancy-related questions used in their clinical trials. Appendix B. Representative citations for authors contributing questionnaire items or with published questionnaires of interest. Appendix C. Draft EXPECT Questionnaire (after Cognitive Testing). [file 1472-6882-14-39-S1.docx]

Additional file 1: APPENDIX A: *List of researchers responding to requests for contributions of expectancy-related questions used in their clinical trials*

| **Researcher [and # in reference list below]** | **CAM Modality** | **Item(s)/Questionnaires used** |
| --- | --- | --- |
| Berman, Brian [2,28,29,58];  Lao, Lixing [2,28,29] | Acupuncture | Locally developed items on belief in treatment; Modification of Borkovec and Nau credibility questions |
| Cambron, Jerilyn [6] | Chiropractic & Massage | Locally developed items on treatment expectations, confidence in treatment, and belief in treatment |
| Cherkin, Dan ; Sherman, Karen [7,8,43,44] | Acupuncture, Chiropractic, Massage, Yoga | Locally developed item on treatment expectations; Local adaptations and measures from Lao, Berman; some measures from Borkovec and Nau; and locally developed item on general optimism regarding status of pain condition |
| Cohen, Lorenzo [10] | Yoga & Acupuncture | Adaptation of Acupuncture Expectancy Scale [35] and locally developed item on confidence in treatment |
| Evans, Roni [5,14,15] | Chiropractic | Locally developed items on treatment expectations |
| Foster, Nadine [19,20] | Acupuncture | Locally developed scale on treatment expectations |
| Goertz, Christine; Hondras, Maria [21] | Chiropractic | Locally developed item on treatment expectations |
| Haas, Mitch [5,40] | Chiropractic | Locally developed item on confidence in treatment |
| Hurwitz, Eric [11,22] | Chiropractic | Locally developed item on confidence in treatment |
| Irnich, Dominick [17,18,23,31] | Acupuncture | Vincent credibility scale [50], which is an adaptation of Borkovec and Nau for acupuncture |
| Kaptchuk, Ted [9,24,25] | Acupuncture | Adaptation of Borkovec and Nau and locally developed items on confidence in treatment |
| Lamb, Sarah E [26,27] | CBT for LBP | Locally developed question on confidence in treatment |
| Lewith, George [30,32,41,52,53,54,58] | various | Adaptation of Borkovec and Nau |
| Linde, Klaus [31] | Acupuncture | Locally developed items on belief in treatment, treatment expectations, and confidence in treatment; Vincent credibility scale [50], which is an adaptation of Borkovec and Nau for acupuncture |
| MacPherson, Hugh [33,34,45] | Acupuncture | Locally developed items on treatment preference, confidence in treatment, and belief in treatment |
| Mao, Jun [35,36] | Acupuncture | Acupuncture Expectancy Scale (validated in China) |
| Moyer, Christopher [1,37,38,39] | Massage | Attitudes Toward Massage Scale [38] |
| Ritenbaugh, Cheryl [42] | Acupuncture, Chiropractic, Massage, ND care | Locally developed items on treatment expectations |
| Torgerson, David [4,12,16,46] | Yoga | Locally developed item on treatment preference |
| Underwood, Martin [4,16,26,27] | Chiropractic | Locally developed item on treatment expectations |
| Vas, Jorge [47,48,49] | Acupuncture | Adaptation of Borkovec and Nau |
| Wayne, Peter [51] | Acupuncture | Adaptation of Borkovec and Nau |
| White, Peter [41,52,53,54] | Acupuncture | Adaptation of Borkovec and Nau and locally developed items on belief in treatment |
| Williams, Kimberly [55,56,57] | Yoga | Locally developed item on treatment expectations |
| Witt, Claudia [31,58,59] | various | Locally developed items on treatment preference |

Additional file 1: APPENDIX B: *Representative citations for authors contributing questionnaire items or with published questionnaires of interest*

1. Beider S, Moyer CA: **Randomized Controlled Trials of Pediatric Massage: A Review**. *Evid Based Complement Alternat Med* 2007, **4**:23–34.

2. Berman BM, Lao L, Langenberg P, Lee WL, Gilpin AMK, Hochberg MC: **Effectiveness of Acupuncture as Adjunctive Therapy in Osteoarthritis of the Knee A Randomized, Controlled Trial**. *Ann Intern Med* 2004, **141**:901–910.

3. Borkovec TD, Nau SD: **Credibility of analogue therapy rationales**. *J Behav Ther Exp Psychiatry* 1972, **3**:257–260.

4. Brealey S, Burton K, Coulton S, Farrin A, Garratt A, Harvey E, Letley L, Martin J, Klaber M, Russell I, Torgerson D, Underwood M, Vickers M, Whyte K, Williams M, null: **UK Back pain Exercise And Manipulation (UK BEAM) trial--national randomised trial of physical treatments for back pain in primary care: objectives, design and interventions [ISRCTN32683578].** *BMC Health Serv Res* 2003, **3**:16.

5. Bronfort G, Haas M, Evans R, Kawchuk G, Dagenais S: **Evidence-informed management of chronic low back pain with spinal manipulation and mobilization**. *Spine J* 2008, **8**:213–225.

6. Cambron JA, Gudavalli MR, Hedeker D, McGregor M, Jedlicka J, Keenum M, Ghanayem AJ, Patwardhan AG, Furner SE: **One-Year Follow-Up of a Randomized Clinical Trial Comparing Flexion Distraction with an Exercise Program for Chronic Low-Back Pain**. *J Altern Complement Med* 2006, **12**:659–668.

7. Cherkin DC, Eisenberg D, Sherman KJ, et al: **Randomized trial comparing traditional chinese medical acupuncture, therapeutic massage, and self-care education for chronic low back pain**. *Arch Intern Med* 2001, **161**:1081–1088.

8. Cherkin DC, Sherman KJ, Avins AL, et al: **A randomized trial comparing acupuncture, simulated acupuncture, and usual care for chronic low back pain**. *Arch Intern Med* 2009, **169**:858–866.

9. Coeytaux RR, Kaufman JS, Kaptchuk TJ, Chen W, Miller WC, Callahan LF, Mann JD: **A Randomized, Controlled Trial of Acupuncture for Chronic Daily Headache**. *Headache*2005, **45**:1113–1123.

10. Cohen L, Warneke C, Fouladi RT, Rodriguez MA, Chaoul-Reich A: **Psychological adjustment and sleep quality in a randomized trial of the effects of a Tibetan yoga intervention in patients with lymphoma**. *Cancer* 2004, **100**:2253–2260.

11. Coulter ID, Hurwitz EL, Adams AH, Genovese BJ, Hays R, Shekelle PG: **Patients using chiropractors in North America: who are they, and why are they in chiropractic care?** *Spine* 2002, **27**:291–296; discussion 297–298.

12. Cox H, Tilbrook H, Aplin J, Chuang L-H, Hewitt C, Jayakody S, Semlyen A, Soares MO, Torgerson D, Trewhela A, Watt I, Worthy G: **A pragmatic multi-centred randomised controlled trial of yoga for chronic low back pain: Trial protocol**. *Complement Ther Clin Pract* 2010, **16**:76–80.

13. Devilly GJ, Borkovec TD: **Psychometric properties of the credibility/expectancy questionnaire**. *J Behav Ther Exp Psychiatry* 2000, **31**:73–86.

14. Evans R, Bronfort G, Bittell S, Anderson AV: **A pilot study for a randomized clinical trial assessing chiropractic care, medical care, and self-care education for acute and subacute neck pain patients**. *J Manipulative Physiol Ther* 2003, **26**:403–411.

15. Evans R, Bronfort G, Nelson B, Goldsmith CH: **Two-year follow-up of a randomized clinical trial of spinal manipulation and two types of exercise for patients with chronic neck pain**. *Spine* 2002, **27**:2383–2389.

16. Farrin A, Russell I, Torgerson D, Underwood M: **Differential recruitment in a cluster randomized trial in primary care: the experience of the UK Back pain, Exercise, Active management and Manipulation (UK BEAM) feasibility study**. *Clin Trials* 2005, **2**:119–124.

17. Fleckenstein J, Lill C, Lüdtke R, Gleditsch J, Rasp G, Irnich D: **A Single Point Acupuncture Treatment at Large Intestine Meridian: A Randomized Controlled Trial in Acute Tonsillitis and Pharyngitis**. *Clin J Pain* 2009, **25**:624–631.

18. Fleckenstein J, Raab C, Gleditsch J, Ostertag P, Rasp G, Stör W, Irnich D: **Impact of Acupuncture on Vasomotor Rhinitis: A Randomized Placebo-Controlled Pilot Study**. *J Altern Complement Med* 2009, **15**:391–398.

19. Foster NE, Thompson KA, Baxter GD, Allen JM: **Management of nonspecific low back pain by physiotherapists in Britain and Ireland : A descriptive questionnaire of current clinical practice**. *Spine* , **24**:1332–1342.

20. Foster NE, Bishop A, Thomas E, Main C, Horne R, Weinman J, Hay E: **Illness perceptions of low back pain patients in primary care: What are they, do they change and are they associated with outcome?** *Pain* 2008, **136**:177–187.

21. Hondras MA, Long CR, Cao Y, Rowell RM, Meeker WC: **A Randomized Controlled Trial Comparing 2 Types of Spinal Manipulation and Minimal Conservative Medical Care for Adults 55 Years and Older With Subacute or Chronic Low Back Pain**. *J Manipulative Physiol Ther* 2009, **32**:330–343.

22. Hurwitz EL, Carragee EJ, van der Velde G, Carroll LJ, Nordin M, Guzman J, Peloso PM, Holm LW, Côté P, Hogg-Johnson S, Cassidy JD, Haldeman S: **Treatment of Neck Pain: Noninvasive Interventions: Results of the Bone and Joint Decade 2000–2010 Task Force on Neck Pain and Its Associated Disorders**. *J Manipulative Physiol Ther* 2009, **32**(2, Supplement):S141–S175.

23. Irnich D: **Randomised trial of acupuncture compared with conventional massage and “sham” laser acupuncture for treatment of chronic neck pain Commentary: Controls for acupuncture---can we finally see the**. *BMJ* 2001, **322**:1574–1574.

24. Kaptchuk TJ, Kelley JM, Conboy LA, Davis RB, Kerr CE, Jacobson EE, Kirsch I, Schyner RN, Nam BH, Nguyen LT, Park M, Rivers AL, McManus C, Kokkotou E, Drossman DA, Goldman P, Lembo AJ: **Components of placebo effect: randomised controlled trial in patients with irritable bowel syndrome**. *BMJ* 2008, **336**:999–1003.

25. Kaptchuk TJ, Friedlander E, Kelley JM, Sanchez MN, Kokkotou E, Singer JP, Kowalczykowski M, Miller FG, Kirsch I, Lembo AJ: **Placebos without Deception: A Randomized Controlled Trial in Irritable Bowel Syndrome**. *PLoS ONE* 2010, **5**:e15591.

26. Lamb SE, Hansen Z, Lall R, Castelnuovo E, Withers EJ, Nichols V, Potter R, Underwood MR: **Group cognitive behavioural treatment for low-back pain in primary care: a randomised controlled trial and cost-effectiveness analysis**. *The Lancet* 13, **375**:916–923.

27. Lamb SE, Lall R, Hansen Z, Withers EJ, Griffiths FE, Szczepura A, Barlow J, Underwood MR, $author.lastName $author firstName: **Design considerations in a clinical trial of a cognitive behavioural intervention for the management of low back pain in primary care: Back Skills Training Trial**. *BMC Musculoskelet Disord* 2007, **8**:14.

28. Lao L, Bergman S, Langenberg P, Wong RH, Berman B: **Efficacy of Chinese acupuncture on postoperative oral surgery pain**. *Oral Surg Oral Med Oral Pathol Oral RadiolEndod* 1995, **79**:423–428.

29. Lao L, Bergman S, Hamilton GR, Langenberg P, Berman B: **Evaluation of acupuncture for pain control after oral surgery: A placebo-controlled trial**. *Arch Otolaryngol Head Neck Surg* 1999, **125**:567–572.

30. Lewith GT, Field J, Machin D: **Acupuncture compared with placebo in post-herpetic pain**. *Pain* 1983, **17**:361–368.

31. Linde K, Streng A, Hoppe A, Brinkhaus B, Witt CM, Hammes M, Irnich D, Hummelsberger J, Willich SN, Melchart D: **Treatment in a Randomized Multicenter Trial of Acupuncture for Migraine (ART Migraine)**. *Forsch Komplementärmed* 2006, **13**:101–108.

32. Little P, Lewith G, Webley F, Evans M, Beattie A, Middleton K, Barnett J, Ballard K, Oxford F, Smith P, Yardley L, Hollinghurst S, Sharp D: **Randomised controlled trial of Alexander technique lessons, exercise, and massage (ATEAM) for chronic and recurrent back pain**. *BMJ* 2008.

33. MacPherson H, Thorpe L, Thomas K: **Beyond Needling—Therapeutic Processes in Acupuncture Care: A Qualitative Study Nested Within a Low-Back Pain Trial**. *J Altern Complement Med* 2006, **12**:873–880.

34. MacPherson H, Thorpe L, Thomas K, Campbell M: **Acupuncture for low back pain: traditional diagnosis and treatment of 148 patients in a clinical trial**. *Complement Ther Med* 2004, **12**:38–44.

35. Mao JJ, Armstrong K, Farrar JT, Bowman MA: **Acupuncture Expectancy Scale: Development and Preliminary Validation in China**. *EXPLORE*2007, **3**:372–377.

36. Mao JJ, Xie SX, Bowman MA: **Uncovering the expectancy effect: the validation of Acupuncture Expectancy Scale**. *Altern Ther Health Med* 2010, **16**:22–27.

37. Moyer CA, Donnelly MPW, Anderson JC, Valek KC, Huckaby SJ, Wiederholt DA, Doty RL, Rehlinger AS, Rice BL: **Frontal Electroencephalographic Asymmetry Associated With Positive Emotion Is Produced by Very Brief Meditation Training**. *Psycho Sci* 2011, **22**:1277–1279.

38. Moyer CA, Rounds J: **The attitudes toward massage (ATOM) scale: Reliability, validity, and associated findings**. *J Bodyw Mov Ther* 2009, **13**:22–33.

39. Moyer CA, Rounds J, Hannum JW: **A Meta-Analysis of Massage Therapy Research**. *Psychol Bul* 2004, **130**:3–18.

40. Nyiendo J, Haas M, Goldberg B, Lloyd C: **A descriptive study of medical and chiropractic patients with chronic low back pain and sciatica: Management by physicians (practice activities) and patients (self-management)**. *J Manipulative Physiol Ther* 2001, **24**:543–551.

41. Pariente J, White P, Frackowiak RSJ, Lewith G: **Expectancy and belief modulate the neuronal substrates of pain treated by acupuncture**. *NeuroImage* 2005, **25**:1161–1167.

42. Ritenbaugh C, Hammerschlag R, Dworkin SF, Aickin MG, Mist SD, Elder CR, Harris RE: **Comparative Effectiveness of Traditional Chinese Medicine and Psychosocial Care in the Treatment of Temporomandibular Disorders–Associated Chronic Facial Pain**.  *J Pain* 2012, **13**:1075–1089.

43. Sherman KJ, Cherkin DC, Connelly MT, Erro J, Savetsky JB, Davis RB, Eisenberg DM: **Complementary and alternative medical therapies for chronic low back pain: What treatments are patients willing to try?** *BMC Complement Altern Med* 2004, **4**:9.

44. Sherman KJ, Cherkin DC, Ichikawa L, Avins AL, Delaney K, Barlow WE, Khalsa PS, Deyo RA: **Treatment Expectations and Preferences as Predictors of Outcome of Acupuncture for Chronic Back Pain**. *Spine (Phila Pa 1976)* 2010, **35**:1471–1477.

45. Thomas KJ, MacPherson H, Thorpe L, Brazier J, Fitter M, Campbell MJ, Roman M, Walters SJ, Nicholl J: **Randomised controlled trial of a short course of traditional acupuncture compared with usual care for persistent non-specific low back pain**. *BMJ* 2006, **333**:623.

46. Tilbrook HE, Cox H, Hewitt CE, Kang’ombe AR, Chuang L-H, Jayakody S, Aplin JD, Semlyen A, Trewhela A, Watt I, Torgerson DJ: **Yoga for Chronic Low Back PainA Randomized Trial**. *Ann Intern Med* 2011, **155**:569–578.

47. Vas J, Ortega C, Olmo V, Perez-Fernandez F, Hernandez L, Medina I, Seminario JM, Herrera A, Luna F, Perea-Milla E, Mendez C, Madrazo F, Jimenez C, Ruiz MA, Aguilar I: **Single-point acupuncture and physiotherapy for the treatment of painful shoulder: a multicentre randomized controlled trial**. *Rheumatology* 2008, **47**:887–893.

48. Vas J, Mendez C, Perea-Milla E, Vega E, Panadero MD, Leon JM, Borge MA, Gaspar O, Sanchez-Rodriguez F, Aguilar I, Jurado R: **Acupuncture as a complementary therapy to the pharmacological treatment of osteoarthritis of the knee: randomised controlled trial**. *BMJ* 2004, **329**:1216.

49. Vas J, Perea-Milla E, Méndez C, Navarro CS, León Rubio JM, Brioso M, Obrero IG: **Efficacy and safety of acupuncture for chronic uncomplicated neck pain: A randomised controlled study**. *Pain* 2006, **126**:245–255.

50. Vincent C: **Credibility assessment in trials of acupuncture**. *Complement Med Res* 1990, **4**:8–11.

51. Wayne PM, Kerr CE, Schnyer RN, Legedza ATR, Savetsky-German J, Shields MH, Buring JE, Davis RB, Conboy LA, Highfield E, Parton B, Thomas P, Laufer MR: **Japanese-Style Acupuncture for Endometriosis-Related Pelvic Pain in Adolescents and Young Women: Results of a Randomized Sham-Controlled Trial**. *J Pediatr Adolesc Gyneco* 2008, **21**:247–257.

52. White P, Bishop FL, Prescott P, Scott C, Little P, Lewith G: **Practice, practitioner, or placebo? A multifactorial, mixed-methods randomized controlled trial of acupuncture**. *Pain* 2012, **153**:455–462.

53. White P, Lewith G, Hopwood V, Prescott P: **The placebo needle, is it a valid and convincing placebo for use in acupuncture trials? A randomised, single-blind, cross-over pilot trial**. *Pain* 2003, **106**:401–409.

54. White P, Lewith G, Prescott P, Conway J: **Acupuncture versus Placebo for the Treatment of Chronic Mechanical Neck PainA Randomized, Controlled Trial**. *Ann Intern Med* 2004, **141**:911–919.

55. Williams K, Abildso C, Steinberg L, Doyle E, Epstein B, Smith D, Hobbs G, Gross R, Kelley G, Cooper L: **Evaluation of the Effectiveness and Efficacy of Iyengar Yoga Therapy on Chronic Low Back Pain:** *Spine* 2009, **34**:2066–2076.

56. Williams K, Steinberg L, Petronis J: **Therapeutic Application of Iyengar Yoga for Healing Chronic Low Back Pain**. *Int J Yoga Therap* 2003, **13**:55–67.

57. Williams KA, Petronis J, Smith D, Goodrich D, Wu J, Ravi N, Doyle Jr EJ, Gregory Juckett R, Munoz Kolar M, Gross R, Steinberg L: **Effect of Iyengar yoga therapy for chronic low back pain**. *Pain* 2005, **115**:107–117.

58. Witt CM, Chesney M, Gliklich R, Green L, Lewith G, Luce B, McCaffrey A, Rafferty Withers S, Sox HC, Tunis S, Berman BM: **Building a Strategic Framework for Comparative Effectiveness Research in Complementary and Integrative Medicine**. *Evid Based Complement Alternat Med* 2012, **2012**.

59. Witt CM, Lüdtke R, Mengler N, Willich SN: **How healthy are chronically ill patients after eight years of homeopathic treatment? – Results from a long term observational study**. *BMC Public Health* 2008, **8**:413.

Additional file 1: *APPENDIX C: DRAFT EXPECT QUESTIONNAIRE (AFTER COGNITIVE TESTING)*

*(Telephone version)*

| The next series of questions is about the effects that **[INSERT TREATMENT MODALITY]** may have on your back pain and on how back pain impacts your life. In each case, the question is asking about the results at the end of the treatment period. | |
| --- | --- |
| **Back Pain Questions:** | |
|  | How much change do you hope for in your back pain? Please answer on a scale of 0 to 10, where 0 is “no change or worse” and 10 is “complete relief”. |
|  | 0 1 2 3 4 5 6 7 8 9 10  **No Change/Worse Complete relief** |
|  | How much change do you realistically expect in your back pain? Please answer on a scale of 0 to 10, where 0 is “no change or worse” and 10 is “complete relief”. |
|  | 0 1 2 3 4 5 6 7 8 9 10  **No Change/Worse Complete relief** |
| **Impact of back pain on life questions:** | |
|  | How much change do you hope for in the impact of back pain on your life? Please answer on a scale of 0 to 10, where 0 is “no change or worse” and 10 is “pain no longer impacts my life”. |
|  | 0 1 2 3 4 5 6 7 8 9 10  **No Change/Worse Back pain no longer impacts my life** |
|  | How much change do you realistically expect in the impact of back pain on your life? Please answer on a scale of 0 to 10, where 0 is “no change or worse”, and 10 is “pain no longer impacts my life”. |
|  | 0 1 2 3 4 5 6 7 8 9 10  **No Change/Worse Back pain no longer impacts my life** |
| **Sleep/Mood/Energy:**  The next set of questions asks about areas of your life such as sleep, mood, and energy. If any of these questions are not relevant for you because back pain does not impact that area of your life, please answer Not Applicable. | |
|  | How much change do you realistically expect in your *back-related* *sleep problems?* Please answer on a scale of 0 to 10, where 0 is “no change or worse”, and 10 is back “pain no longer affects my sleep”. If back pain does not impact your sleep, please choose “not applicable”. |
|  | 0 1 2 3 4 5 6 7 8 9 10  **No Change/Worse Back pain no longer affects my sleep**  □ Not Applicable |
|  | How much change do you realistically expect in your *mood or irritability?* Please answer on a scale of 0 to 10, where 0 is “no change or worse”, and 10 is “back pain no longer affects my mood”. Or you may choose “NA”, not applicable. |
|  | 0 1 2 3 4 5 6 7 8 9 10  **No Change/Worse Back pain no longer affects my mood or irritability**  □ Not Applicable |
|  | How much change do you realistically expect in your *energy?*  Please answer on a scale of 0 to 10, where 0 is “no change or worse”, and 10 is “back pain no longer affects my energy”. Or you may choose “NA”, not applicable. |
|  | 0 1 2 3 4 5 6 7 8 9 10  **No Change/Worse Back pain no longer affects my energy**  □ Not Applicable |
|  |  |
| ***Coping Question:***  The next question is about your expectations about coping with back pain. | |
|  | How much improvement in your ability to cope with back pain do you realistically expect as a result of **[INSERT TREATMENT MODALITY]**? Please answer on a scale of 0 to 10, where 0 is “no improvement” and 10 is “extreme improvement”. |
|  | 0 1 2 3 4 5 6 7 8 9 10  **No improvement Extreme Improvement** |
|  |  |
| **Limitations due to back pain Questions:**  The following questions are about effects that **[INSERT TREATMENT MODALITY]** may have on your physical limitations due to back pain. In each case, the question is asking about the results at the end of the treatment period. If these questions are not relevant for you because you do not have any physical limitations due to back pain, please choose Not Applicable. | |
|  | How much change do you hope you will have in your back pain-related physical limitations? Please answer on a scale of 0 to 10, where 0 is “no change or worse”, and 10 is “limitations completely resolved”. Or you may choose “NA”, not applicable. |
|  | 0 1 2 3 4 5 6 7 8 9 10  **No Change/Worse Limitations completely resolved**  □ Not Applicable |
|  | How much change do you realistically expect in your back pain-related physical limitations? Please answer on a scale of 0 to 10, where 0 is “no change or worse”, and 10 is “limitations completely resolved”. Or you may choose “NA”, not applicable. |
|  | 0 1 2 3 4 5 6 7 8 9 10  **No Change/Worse Limitations completely resolved**  □ Not Applicable |
|  |  |
| **Impact of back pain on specific areas of life questions:**  The next questions to ask about the effects that **[INSERT TREATMENT MODALITY]** may have on the impact of back pain on specific areas of your life. In each case, the question is asking about the results at the end of the treatment period. If any of these questions are not relevant for you because back pain does not impact that area of your life, please choose Not Applicable. | |
|  |  |
|  | How much change do you realistically expect in the impact of back pain on your **work, including housework?** Please answer on a scale of 0 to 10, where 0 is “no change or worse” and 10 is “back pain no longer impacts my **work”**, including housework. Or you may choose “NA”, not applicable if back pain does not impact your housework now. |
|  | 0 1 2 3 4 5 6 7 8 9 10  **No Change/Worse Back pain no longer impacts my work**  □ Not Applicable |
|  | How much change do you realistically expect in the impact of back pain on your **social and recreational activities?** Please answer on a scale of 0 to 10, where 0 is “no change or worse” and 10 is “back pain no longer impacts my **social and recreational activities”.** Or you may choose “NA”, not applicable if back pain does not impact your social and recreational activities now. |
|  | 0 1 2 3 4 5 6 7 8 9 10  **No Change/Worse Back pain no longer impacts my social and recreational activities**  □ Not Applicable |
|  | How much change do you realistically expect in the impact of back pain on your **daily activities?** Please answer on a scale of 0 to 10, where 0 is “no change or worse” and 10 is “back pain no longer impacts my **daily activities”**. Or you may choose “NA”, not applicable if back pain does not impact your daily activities now. |
|  | 0 1 2 3 4 5 6 7 8 9 10  **No Change /Worse Back pain no longer impacts my daily activities**  □ Not Applicable |
|  | How much change do you realistically expect in the impact of back pain on your **relationships with family and friends?** Please answer on a scale of 0 to 10, where 0 is “no change or worse” and 10 is “back pain no longer impacts my relationships with family and friends”. Or you may choose “NA”, not applicable if back pain does not impact your relationships with family and friends now. |
|  | 0 1 2 3 4 5 6 7 8 9 10  **No Change /Worse Back pain no longer impacts my relationships**  □ Not Applicable |
|  | |
| **Long-Term Outcome questions:**  The previous questions asked about your hopes and expectations for change in your back pain at the completion of treatment. Now we shift to questions about your back pain **ONE YEAR** from now. As you answer the following questions, please take into account the course of treatment you plan to complete, plus any self-care or other health care you think you might do in that time period. | |
|  | How much change do you hope for in your back pain one year from now? Please answer on a scale of 0 to 10, where 0 is “no change or worse” and 10 is “complete relief”. |
|  | 0 1 2 3 4 5 6 7 8 9 10  **No Change/Worse Complete relief** |
|  | How much change do you realistically expect in your back pain one year from now? Please answer on a scale of 0 to 10, where 0 is “no change or worse” and 10 is “complete relief”. |
|  | 0 1 2 3 4 5 6 7 8 9 10  **No Change/Worse Complete relief** |
|  | How much change do you hope for in the impact of back pain on your life one year from now? Please answer on a scale of 0 to 10, where 0 is “no change or worse”, and 10 is “back pain no longer impacts my life”. |
|  | 0 1 2 3 4 5 6 7 8 9 10  **No Change /Worse Back pain no longer impacts my life** |
|  | How much change do you realistically expect in the impact of back pain on your life one year from now? Please answer on a scale of 0 to 10, where 0 is “no change or worse”, and 10 is “back pain no longer impacts my life”. |
|  | 0 1 2 3 4 5 6 7 8 9 10  **No Change /Worse Back pain no longer impacts my life** |
|  |  |
